# Enhancement of direct interspecies electron transfer and methane production by co-culture of dual *Methanosarcina* species and *Geobacter metallireducens*

**DOI:** 10.3389/fmicb.2025.1604265

**Published:** 2025-08-05

**Authors:** Lu Liu, Pengsong Li, He Dong, Chuanqi Liu, Haoyong Li, Zihao Ma, Ruoyu Li, Yan Dang

**Affiliations:** ^1^Beijing Key Laboratory for Source Control Technology of Water Pollution, Engineering Research Center for Water Pollution Source Control and Eco-Remediation, College of Environmental Science and Engineering, Beijing Forestry University, Beijing, China; ^2^Beijing Hepingjie No.1 Middle School, Beijing, China; ^3^Laboratory and Facility Management Office, Beijing Forestry University, Beijing, China

**Keywords:** direct interspecies electron transfer, *Methanosarcina*, *G. metallireducens*, conductive materials, anaerobic digestion, microbial synergy

## Abstract

Anaerobic digestion is a key technology for converting organic waste into methane, offering significant potential for renewable energy production and waste management. While the addition of conductive materials has been shown to improve direct interspecies electron transfer (DIET), their application faces challenges like biofouling, environmental risks, and increased operational costs. This study investigated the effects of co-culturing dual *Methanosarcina* (*Methanosarcina barkeri* and *Methanosarcina acetivorans*) and *Geobacter metallireducens* (DM-G) to enhance DIET and methane production without the addition of exogenous conductive materials. The performance of the DM-G co-culture system was comparable to that of the conductive material-amended single *Methanosarcina* and *G. metallireducens* (SM-G) co-culture systems, achieving a maximum methane concentration of 19.5 mM, following the consumption of 15.2 mM ethanol in the 1:1:1 biomass ratio system. This corresponds to a 3.8-fold increase over the SM-G co-culture system with *M. barkeri* and *G. metallireducens*, and a 3.0-fold increase over that with *M. acetivorans* and *G. metallireducens*. Transcriptomic analysis showed that in the DM-G co-culture system, *M. barkeri* up-regulated key genes related to methane metabolism and acetate utilization, while the core methanogenic pathway of *M. acetivorans* was down-regulated, but it could still effectively utilize the electron transfer pathway, indicating metabolic complementarity. These findings propose a novel strategy for enhancing DIET-driven methanogenesis through synergistic microbial consortia, advancing scalable, low-cost bioenergy solutions for organic waste valorization.

## 1 Introduction

Against the backdrop of the global clean energy transition, anaerobic digestion (AD), as a core technology for organic waste valorization and bioenergy production, plays a critical role in achieving the “double carbon” goals. However, anaerobic treatment of high-strength organic wastewater commonly faces imbalances between acidogenesis and methanogenesis, posing a risk of system acidification due to the accumulation of volatile fatty acids (VFA) (Van Kessel and Russell, 1996; Yu and Fang, 2003). Methanogens exhibit limited substrate utilization (e.g., reliance on inorganic CO_2_, formate, or methanol), relying on hydrogen as an electron carrier for methanogenesis. This process is constrained by diffusion distances and inevitably involves H2 loss during diffusion (de Bok et al., 2004), which collectively hinder electron transfer efficiency and utilization, thus limiting the efficient conversion of organic matter to methane in high-strength wastewater AD systems.

In 2010, scholars such as Summers (Summers et al., 2010) first verified the existence of DIET in the co-culture system of *G. metallireducens* and *Geobacter sulfurreducens*. The research pointed out that even if the hydrogenase of *G. sulfurreducens* was knocked out, making it lose the ability to utilize H_2_, it could still form a stable symbiotic relationship with *G. metallireducens*, indicating that the electron transfer between the two was not completely mediated by H_2_. In addition, the DIET mechanism may also exist in the methanogenic flora. Morita et al. (2011) found in the anaerobic sludge reactor of brewery waste that granular sludge could form highly conductive aggregates through DIET, and its electrical conductivity was even three times that of the *Geobacter* co-culture system.

In addition to the microbial structure itself, exogenous conductive materials can also significantly promote the DIET process (Kato et al., 2012b). Previous studies have shown that the supplementation of carbon-based (such as granular activated carbon, GAC) and iron-based conductive materials (such as magnetite) can serve as an electron conduction channel between *Methanosarcina* and electroactive bacteria such as *G. metallireducens*, thus enhancing DIET and further increasing methane production. Experiments have found that the addition of biochar can significantly increase the methane production rate of the co-culture system of *G. metallireducens* and *Methanosarcina barkeri* (Chen et al., 2014). Similarly, the addition of GAC can enhance the electron exchange between methanogens and syntrophic bacteria, thus accelerating methane production (Liu et al., 2014) In addition, Kato et al. (2012a) proposed that the introduction of iron oxide nanoparticles (such as magnetite) can enrich the methanogenic flora and enhance interspecies interactions, further optimizing the system performance. Although conductive materials have been proven to enhance DIET by simulating the electron transfer interface, their practical applications face problems such as efficiency attenuation caused by bio-attachment, environmental release risks, and high costs. There is an urgent need to develop sustainable solutions relying on the metabolic characteristics of microorganisms themselves.

Methanosarcina species can be classified into two methanogenic metabolic types: Type I, exemplified by *M. barkeri*, possesses hydrogenase activity, optimizes electron utilization via hydrogen cycling, and gains metabolic dominance in high electron flux environments. Type II, exemplified by *Methanosarcina acetivorans* relies on acetate as a carbon source, acquiring electrons through transmembrane cytochrome complexes, and depends on the Rnf complex for energy metabolism due to the absence of hydrogenase (Zhou et al., 2021). The differences in electron transport pathways and energy metabolic strategies between the two groups provide a theoretical basis for constructing functionally complementary co-culture systems. However, how their metabolic complementarity regulates electron allocation in DIET processes and enhances methane synthesis efficiency still requires in-depth investigation.

In this study, we investigated the potential of co-culturing *M. barkeri* and *M. acetivorans* in combination with *G. metallireducens* to enhance DIET and methane production, without the need for additional conductive materials. We aim to elucidate the underlying mechanisms of electron transfer and explore the feasibility of a microbial-based solution to improve AD processes, offering a more cost-effective and sustainable alternative for bioenergy production.

## 2 Materials and methods

### 2.1 Microbial strains, media, and culture conditions

The study used two types of *Methanosarcina*, namely Type I *M. barkeri* and Type II *M. acetivorans*, as well as *G. metallireducens*, all of which were from the laboratory’s strain collection. The experiment was carried out under strict anaerobic culture conditions. The gas phase was maintained at an N_2_-CO_2_ ratio of 80:20, and the cultures were conducted in serum bottles sealed with thick butyl rubber stoppers.

Both types of *Methanosarcina* were grown in modified DSM 120 medium at a constant temperature of 37°C. The modifications to DSM 120 were as follows: 0.5 mM sulfide, 1 mM cysteine, 0.22 g/L CaCl_2_⋅2H_2_O, no yeast extract, no Casitone, no resazurin, and 2 g/L NaHCO_3_ (Rotaru et al., 2014). The medium for Type I *M. barkeri* contained 0.2% NaCl, while the medium for Type II *M. acetivorans* contained 0.5% NaCl. The substrates for the single cultures of the two bacteria were 100 mM methanol and 50 mM sodium acetate.

*Geobacter metallireducens* was typically cultured in ferric citrate medium (FC) supplemented with 10 mM acetate. The basic components of the medium were: 2.5 g/L NaHCO_3_, 0.1 g/L KCl, 0.25 g/L NH_4_Cl, 0.6 g/L sodium dihydrogen phosphate monohydrate, as well as vitamin and trace element mixtures, with 13.7 g/L ferric citrate as the electron acceptor (Lovley et al., 1993; Tremblay et al., 2011). However, before coculturing with *Methanosarcina*, *G. metallireducens* needed to be sub-cultured 3–5 times (with an inoculum size of 10% each time) in ferric citrate medium where 20 mM ethanol was used to replace acetate, to fully adapt to ethanol as the substrate.

### 2.2 Establishment of co-culture systems

Two distinct co-culture systems were constructed to investigate DIET between *Methanosarcina* species and *G. metallireducens* under different biomass ratios: (1) single *Methanosarcina* with *G. metallireducens* (SM-G) co-culture systems, including *M. acetivorans* with *G. metallireducens* and *M. barkeri* with *G. metallireducens*, which were abbreviated as M. a+G. m and M. b+G. m, respectively; (2) dual *Methanosarcina* with *G. metallireducens* (DM-G) co-culture systems, consisting of *M. acetivorans*, *M. barkeri*, *G. metallireducens* at a biomass ratio of 1:1:1, which was abbreviated as M. a+M. b+G. m (1+1+1) and the other group is inoculated at a biomass ratio of 1:1:2, which was abbreviated as M. a+M. b+G. m (1+1+2).

All the co-culture experiments adopted DSM 120 medium, which contains specific components per liter. After sterilization, anaerobic and sterile solutions such as sodium bicarbonate need to be added (Holmes et al., 2021). To ensure the normal growth of *G. metallireducens* in the co-culture system, the salinity of the medium for pure culture of *M. acetivorans* was reduced from 0.5% to 0.2% (Rotaru et al., 2014; Holmes et al., 2018). In addition, *G. metallireducens* preconditioning involved three successive subcultures in ferric citrate medium with 5%–10% (v/v) inoculum transfer to ensure ethanol substrate adaptation. Under strict anaerobic conditions, when *M. barkeri*, *M. acetivorans* and *G. metallireducens* are all in the logarithmic growth phase (OD_600_≈0.4–0.6), they are respectively, inoculated into DSM 120 medium (10 mL/110 mL anaerobic vial) at a proportion of 30% (v/v) each. Using 20 mM ethanol as the substrate, the cultures are incubated at 30°C for 38–70 days, and three parallels are set for each group of experiments (Liu et al., 2012; Liu et al., 2014). Routine monitoring included headspace gas analysis and optical density measurements to track metabolic activity. All data obtained from the experiments were derived from three parallel groups and subjected to significance analysis using *t*-test.

### 2.3 Preparation and addition of conductive materials

In this experimental study, two conductive materials were used: GAC (particle size range of 2–3 mm, 6 mesh) and nano-magnetite (diameter: 20–50 nm). To ensure the accuracy and reliability of the experimental results and minimize the impact of microorganisms on the surface of GAC on the co-culture system, strict and meticulous pretreatment was carried out on the GAC before addition. First, the GAC was ultrasonically cleaned with acetone for 30 min. This process can effectively remove most of the organic impurities and dust adhering to the surface of GAC. Then, the acetone was poured out. Subsequently, it was ultrasonically cleaned with absolute ethanol for 30 min to further remove the remaining organic components, and then the absolute ethanol was poured out. Finally, the GAC was ultrasonically cleaned with deionized water several times until it was completely odorless, thoroughly removing any potentially remaining organic solution to ensure the purity of GAC. After cleaning, the GAC was placed in an oven at 60°C for drying. Before the sterilization of the medium, according to the experimental design requirements, 25 g/L of GAC with small size of 2–3 mm was added to the anaerobic vials of different SM-G co-culture groups (Liu et al., 2012).

Preparation of nano-magnetite (Lovley et al., 1987): 0.8 M FeCl_3_ and 0.4 M FeCl_2_ were slowly added to a 0.4 M HCl solution. The obtained acidic Fe (II) /Fe (III) solution was slowly added to a vigorously stirred 1.5 M NaOH solution for mixing. After centrifugal purification, it was suspended in deionized water to prepare nano-magnetite (Liu et al., 2014; Holmes et al., 2024). Before sterilizing the medium, 10 mM nano-magnetite was added, respectively. Each SM-G co-culture system with added nano-magnetite was set up with three parallel experimental groups.

### 2.4 Analytical methods

During the experiment, 1 mL syringes were used to sample the headspace gas, which was then injected into a gas chromatograph (Tianmei, GC7900, China) for gas composition detection and analysis. The gas chromatograph is equipped with a flame ionization detector (FID), which is widely used due to its high sensitivity. During the detection process, argon was used as the carrier gas, the injection volume was 1 mL, and the column temperature was 120°C. The determination method referred to the operation instructions of GC7900. Ethanol was determined by a gas chromatograph (Agilent GC 7800A). Its parameters are as follows: the detector is FID, the chromatographic column is Agilent 19095N-123, the detector temperature is 300°C; the hydrogen flow rate is 60 mL/min, the air flow rate is 400 mL/min, and the make-up gas flow rate is 35 mL/min; the injection volume is 1 μL (Dong et al., 2025). A high-performance liquid chromatograph (Scion LC6000) was used to determine the concentration of acetic acid, an intermediate metabolite of ethanol. Chromatographic analysis was carried out using an Aminex HPX-87X column (300 × 7.8 mm). The operating conditions of this liquid chromatograph are as follows: a 0.5 mM dilute sulfuric acid solution was used as the mobile phase, the flow rate was set at 0.6 mL/min, the injection volume was 20 μL, a UV spectrophotometric detector was used, the detection wavelength was 254 nm, and the column temperature was 35°C (Nevin et al., 2008). To ensure the accuracy and repeatability of the determination, the operation instructions of Scion LC6000 were strictly followed.

### 2.5 Transcriptomic analysis

During the logarithmic growth phase, cultures from all co-culture systems and three single-culture strains were harvested for transcriptome analysis. Specifically, when the methane concentration reached approximately 15 mM, the co-culture samples and cells of dual *Methanosarcina* were collected; when the ferrous ion concentration in the *G. metallireducens* culture reached 35 mM, its cells were collected (Holmes et al., 2021). Cell pellets of all samples were obtained by centrifugation at 4000 × *g* for 15 min at 4°C in 50 mL centrifuge tubes. After centrifugation, the pellets were frozen in liquid nitrogen and stored at −80°C until RNA extraction (Holmes et al., 2014).

Total RNA was extracted from the sample pellets following a previously described method (Holmes et al., 2012). Metatranscriptomic pairedend sequencing was performed using Illumina HiSeq × Ten (2 × 150 bp). The raw data were subjected to quality checking with FASTQC, trimming with Trimmomatic (Bolger et al., 2014), and merging with FLASH (Magoč and Salzberg, 2011). Subsequently, ribosomal RNA (rRNA) reads were removed from the library using SortMeRNA (DNASTAR) (Kopylova et al., 2012). The trimmed mRNA reads were aligned to the genomes of *G. metallireducens* (CP000148), *M. barkeri* (WWM608), and *M. acetivorans* (NC_003552) using SeqMan NGen (DNAStar) software. The reads were then normalized using the edgeR package in Bioconductor (Robinson et al., 2009) for differential expression studies.

For another round of transcriptome analysis, early-to-mid stage co-culture cells were collected and centrifuged at 5000 × *g* for 15 min at 4°C. Total RNA was extracted using TRIzol reagent (Invitrogen, California, USA) as previously described. A directional library was prepared using the NEBNext^®^ Ultra II™ Directional RNA Library Prep Kit (New England Biolabs, Beijing, China). Paired end sequencing of mRNA was then carried out using an Illumina HiSeq/MiSeq platform. All raw sequencing data were quality-checked and filtered as described above. Reads matching the 16S and 23S rRNA genes were removed, and the remaining reads were aligned to the published genomes of *G. metallireducens* (e.g., ATCC 53774, or a similar genome if used in your study) and *M. barkeri* (NZ_CP009528.1). Normalization was performed using fragments per kilobase (FPKM) as previously reported (Huang et al., 2022).

### 2.6 16S rRNA gene sequencing for microbial community analysis

Subsequent high-throughput sequencing is required for the DNA extraction of three single bacteria. For archaea and bacteria, the PCR technique is used to amplify their 16S rRNA gene fragments. The primers for archaea and bacteria are (Arch524F/Arch958R) and (338F/806R), respectively (Peiffer et al., 2013). High-throughput sequencing is carried out on the Illumina Hiseq 2000 platform (Illumia, San Diego, USA) by Shanghai Majorbio Bio-pharm Technology Co., Ltd. (Major Biotechnology Co. Ltd.). Following sequencing, sequences were classified into different Operational Taxonomic Units (OTUs) using Pyrosequencing Pipeline software.

### 2.7 Fluorescence in situ hybridization

This study employed triple-probe Fluorescence in situ hybridization (FISH) to analyze the spatial interactions of *M. barkeri*, *M. acetivorans*, and *G. metallireducens* in DM-G co-culture systems. Co-culture samples at the logarithmic growth phase were fixed in 50 mM PIPES buffer (pH = 6.8) containing 0.5% glutaraldehyde and 1% paraformaldehyde for 2 h, followed by sequential ethanol dehydration at 4°C. Tissues were embedded in paraffin, and 4 μm sections were prepared using a microtome. After dewaxing in xylene and rehydration through graded ethanol series, sections were rinsed with DEPC-treated water and PBS. Pre-hybridization was performed at 37°C for 1 h using a hybridization buffer, followed by overnight incubation at 40°C with species-specific probes: FAM-labeled *M. barkeri* probe (5′-GTGCTCCCCCGCCAATTCCT-3′) (Huang et al., 2022), Cy3-labeled *M. acetivorans* probe (5′-GTAGTCCCAGCCGTAAACGA-3′) (Holmes et al., 2021), and Cy5-labeled *G. metallireducens* probe (5′-AGAATCCAAGGACTCCGT-3′) (Nevin and Lovley, 2002). After post-hybridization washing, the sections were imaged using a NIKON ECLIPSE TI confocal microscope at excitation wavelengths of 488 nm (for FAM, emitting green fluorescence), 552 nm (for Cy3, emitting red fluorescence), and 638 nm (for Cy5, emitting far-yellow fluorescence) Additionally, microbial morphology (cocci/rods) was used to validate the species identification.

### 2.8 Morphological analysis of microorganisms

In order to visually present the binding and attachment of *Methanosarcina* and *Geobacter* on conductive materials, as well as the fine morphology of microorganisms, we conducted scanning electron microscopy (SEM) observations on GAC and nano-magnetite, which showed good methane producing effects in the enhanced DIET system.

Before the SEM observation, the conductive materials were pretreated as follows: The conductive materials were taken out from the co-culture system that had been cultured to the late logarithmic growth phase and washed several times with ultrapure water to remove impurities such as leachate and humus. After discarding the supernatant, 2.5% glutaraldehyde (pH = 6.8) was added, and the samples were fixed at 4°C for 1.5 h. The fixed samples were rinsed 3 times with 0.1 M phosphate buffered saline (PBS, pH = 6.8), and then centrifuged at 3000 rpm for 10 min to discard the supernatant. Subsequently, gradient dehydration was carried out with ethanol at concentrations of 50%, 70%, 80% and 90% for 10 min each time, followed by dehydration with 100% ethanol 3 times, 10 min each time. Finally, the samples were placed in a desiccator and dried for 24–48 h (Rotaru et al., 2014). The dried samples were pasted onto the sample stage, coated with a layer of Au film on the surface using an ion sputtering coater, and then observed with an s-4700 scanning electron microscope.

### 2.9 Statistical analysis

Gene Ontology enrichment analysis was performed using Goatools (Gene Ontology ATtainment, v1.2.0). The Rich factor for each GO term was calculated as the ratio of significantly enriched differentially expressed genes (DEGs) within the term to the total number of genes annotated to that term in the reference genome (Background number). A higher Rich factor indicates a greater degree of functional enrichment. Statistical significance was assessed by Fisher’s exact test to generate raw *P*-values, followed by Benjamini-Hochberg (BH) correction for false discovery rate (FDR) control. A lower P-adjust denotes stronger statistical significance. GO terms with adjusted *P*-value (P-adjust) < 0.05 were considered statistically significant.

## 3 Results

### 3.1 Enhanced methane production in DM-G co-culture systems

**FIGURE 1:**
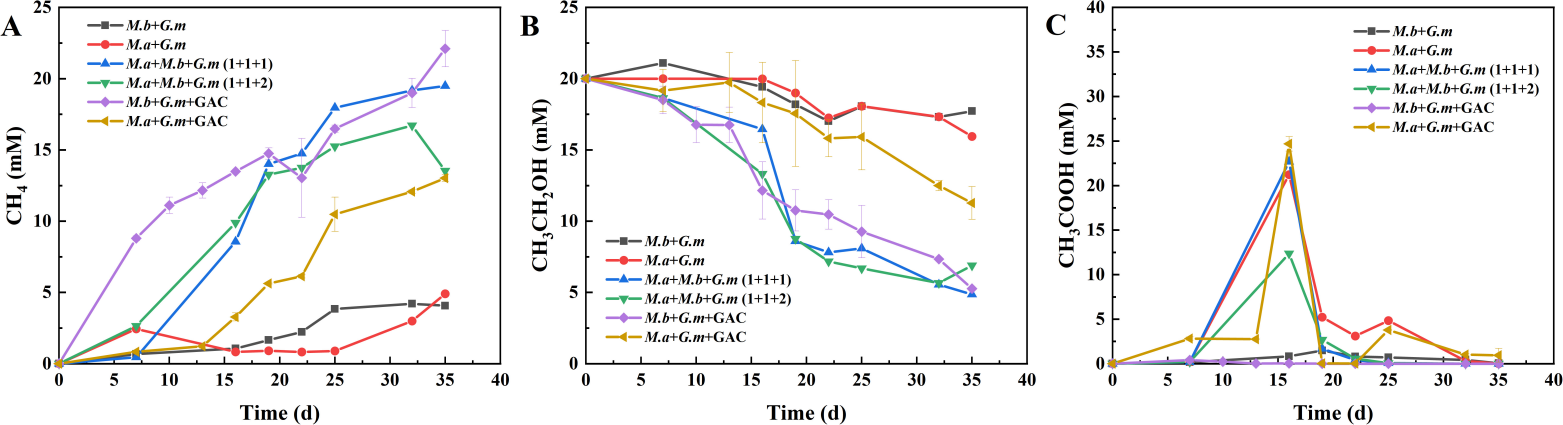
Methane production, ethanol metabolism, and acetic acid metabolism in SM-G and DM-G co-culture systems with or without GAC. **(A)** Methane production. **(B)** Ethanol metabolism. **(C)** Acetic acid metabolism. Data are presented as mean values with standard errors from three replicate cultures. Error bars represent the range of standard errors.

This study examined the synergistic effects of co-culturing *M. barkeri*, *M. acetivorans*, and *G. metallireducens* on methane production. The results showed that methane production was significantly enhanced in DIET co-cultures inoculated with two *Methanosarcina* species and *G. metallireducens* (DM-G) compared to DIET with only one *Methanosarcina* species and *G. metallireducens* (SM-G). Previous research indicated a lag phase of approximately 30–39 days when a single *Methanosarcina* is co-cultured with *G. metallireducens* (Rotaru et al., 2014; Yee and Rotaru, 2020; Holmes et al., 2021), a 35 days lag period was observed for the SM-G co-cultures (Figures 1B, C). However, when both *Methanosarcina* species were cultured together with *G. metallireducens*, methane production started after only 15 days of cultivation. Ethanol was rapidly oxidized to acetate, which reached its highest concentration before being completely converted into methane. After 35 days, in the DM-G co-culture systems of 1:1:1 and 1:1:2, the substrate ethanol was consumed by 15.2 mM and 13.1 mM respectively, the methane concentrations reached 19.5 mM and 16.7 mM. Compared with the co-culture of *M. barkeri* with *G. metallireducens*, they increased by 3.8-fold and 3.1-fold respectively; compared with the co-culture of *M. acetivorans* with *G. metallireducens*, they increased by 3.0-fold and 2.6-fold, respectively (Figure 1A). These results indicate that a synergistic mechanism was occurring that better facilitated DIET.

In this study, GAC and nano-magnetite were added to co-culture systems of *M. barkeri* and *G. metallireducens*, as well as *M. acetivorans* and *G. metallireducens*, with a 35-days observation period. Furthermore, it can be discerned from the SEM that *Methanosarcina* and *Geobacter* are in mutual contact and adhere to the two materials. There are differences that GAC demonstrates a pronounced propensity to promote the close attachment and collaborative behavior of microorganisms. Microbial cells can be deeply embedded within its nanopores, with cells being in close proximity to each other (Supplementary Figures 1A–C). This close-range contact significantly facilitates the direct contact between cells, thereby substantially accelerating the rate of metabolite exchange. Microorganisms attach tightly and in large numbers to the surface of GAC, while on the surface of nano-magnetite, the attachment is relatively uniform and can form a stable biofilm (Liu et al., 2014). While GAC supports the attachment of microbial cells, nano-magnetite, due to its smaller diameter, tends to aggregate bacterial cells, making it difficult for *Methanosarcina* and *Geobacter* to establish direct contact (Supplementary Figures 1D–F). Despite this, nano-magnetite still enhances DIET due to its conductive properties.

Among the four enhanced co-culture systems with conductive materials, GAC had the most significant effect on methane production. In co-cultures of *M. barkeri* and *G. metallireducens*, ethanol consumption reached 14.7 mM, with final methane production peaking at 22.1 mM (Figure 1A). This represents a 4.4-fold increase over controls. Although nano-magnetite also promotes methane generation, the final methane concentration was lower than that achieved with GAC. In this case, 12.0 mM of ethanol was consumed, and the methane concentration reached 18.0 mM, representing a 3.4-fold increase (Supplementary Figure 2A). In the co-culture of *M. acetivorans* and *G. metallireducens*, the addition of GAC enabled the system to consume 8.7 mM of ethanol, with the methane concentration reaching 13.0 mM, a 1.7-fold increase (Figure 1A). Nano-magnetite also significantly enhanced the co-culture system, where 9.2 mM of ethanol was consumed, and 14.5 mM of methane was produced, corresponding to a 2.0-fold increase. Moreover, the ethanol-derived acetate was rapidly converted to methane (Supplementary Figure 2).

In these co-culture systems, ethanol was first metabolized to acetate (Figures 1B, C), which was then quickly converted into methane, with minimal lag time. This suggests that DIET was efficient, and the addition of conductive materials improved ethanol metabolism efficiency, supporting stable microbial interactions (Wu Y. et al., 2020). Both methane production and ethanol consumption followed the chemical equation: 2C_2_H_6_O→3CH_4_ + *CO*_2_. In this experiment, ethanol consumption generally adhered to the theoretical ratio that for every mole of ethanol consumed, 1.5 moles of methane are produced (Rotaru et al., 2014).

Additionally, in the DIET process, *M. acetivorans* produced higher concentrations of acetate than *M. barkeri*. This may be because *M. acetivorans* belongs to Type II *Methanosarcina*, which primarily utilizes acetate and electrons for metabolism. Due to the absence of hydrogenase, *M. acetivorans* has a lower efficiency in utilizing electrons for acetate metabolism, leading to slower acetate oxidation and more accumulation of acetate. In contrast, *M. barkeri* possesses hydrogenase, enabling it to more efficiently accept and utilize electrons transferred from *G. metallireducens*. The presence of hydrogenase accelerates acetate oxidation through the hydrogen cycle, facilitating the rapid conversion of acetate into methane (Zhou et al., 2021). Furthermore, the efficient electron transfer mechanism allows *M. barkeri* to exhibit stronger metabolic activity in high electron flux environments, further enhancing methane production.

Comparative analysis of the two systems demonstrates that the DM-G systems enhanced methane production, efficient electron transfer, and optimized metabolic pathways highlight its advantages over the SM-G systems. From an electron transfer perspective, DM-G system may achieve efficient electron transfer and utilization through different pathways or mechanisms, compensating for the limitations of SM-G system and enhancing the overall DIET efficiency. In terms of metabolic pathways, the synergistic effect of *M. barkeri* and *M. acetivorans* may have created new metabolic pathways or optimized existing ones, allowing ethanol to be converted into methane more quickly and thoroughly, thereby improving substrate utilization efficiency.

### 3.2 Microbial community shifts in DM-G co-culture system

In the DM-G co-culture systems, before inoculation, both *Methanosarcina* and *G. metallireducens* were cultured to the logarithmic growth phase to ensure relatively stable physiological states and similar growth activities. The inoculation volumes of *M. acetivorans* and *M. barkeri* were 30% (v/v), but the volume of *G. metallireducens* was 30% (v/v) and 60% (v/v) in the 1:1:1 and 1:1:2 systems, respectively.

**FIGURE 2:**
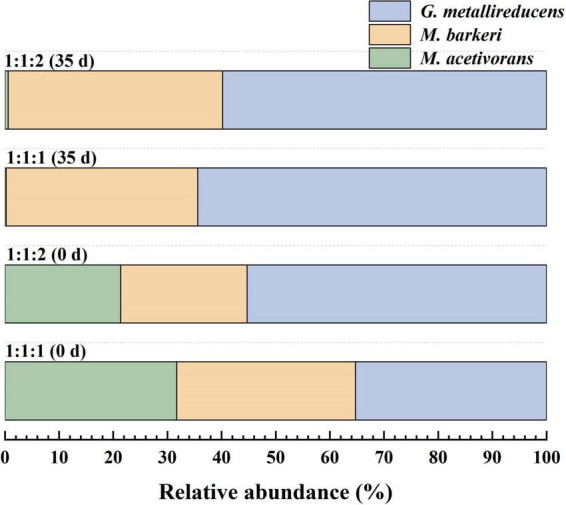
Distribution changes of three strains (*M. barkeri*: *M. acetivorans*: *G. metallireducens*) in two DM-G co-culture systems at the initial stage (0 day) and on the 35th day of cultivation under two inoculation ratios.

Absolute quantification at the Amplicon Sequence Variant (ASV) level was carried out to precisely analyze the relative abundances of the three strains at the initial inoculation stage (Day 0). The results showed that in the 1:1:1 system, the relative abundances of ASV of *M. acetivorans*, *M. barkeri* and *G. metallireducens* were 31.7%, 33.3% and 35.3%, respectively. In the 1:1:2 system, the relative abundances of *M. acetivorans*, *M. barkeri* and *G. metallireducens* were 21.4%, 23.4% and 55.3%, respectively (Figure 2). These results were highly consistent with the theoretical inoculation ratios designed in the experiment (*p* < 0.05), indicating that the sequencing method could accurately reflect the characteristics of the initial microbial community composition.

**FIGURE 3:**
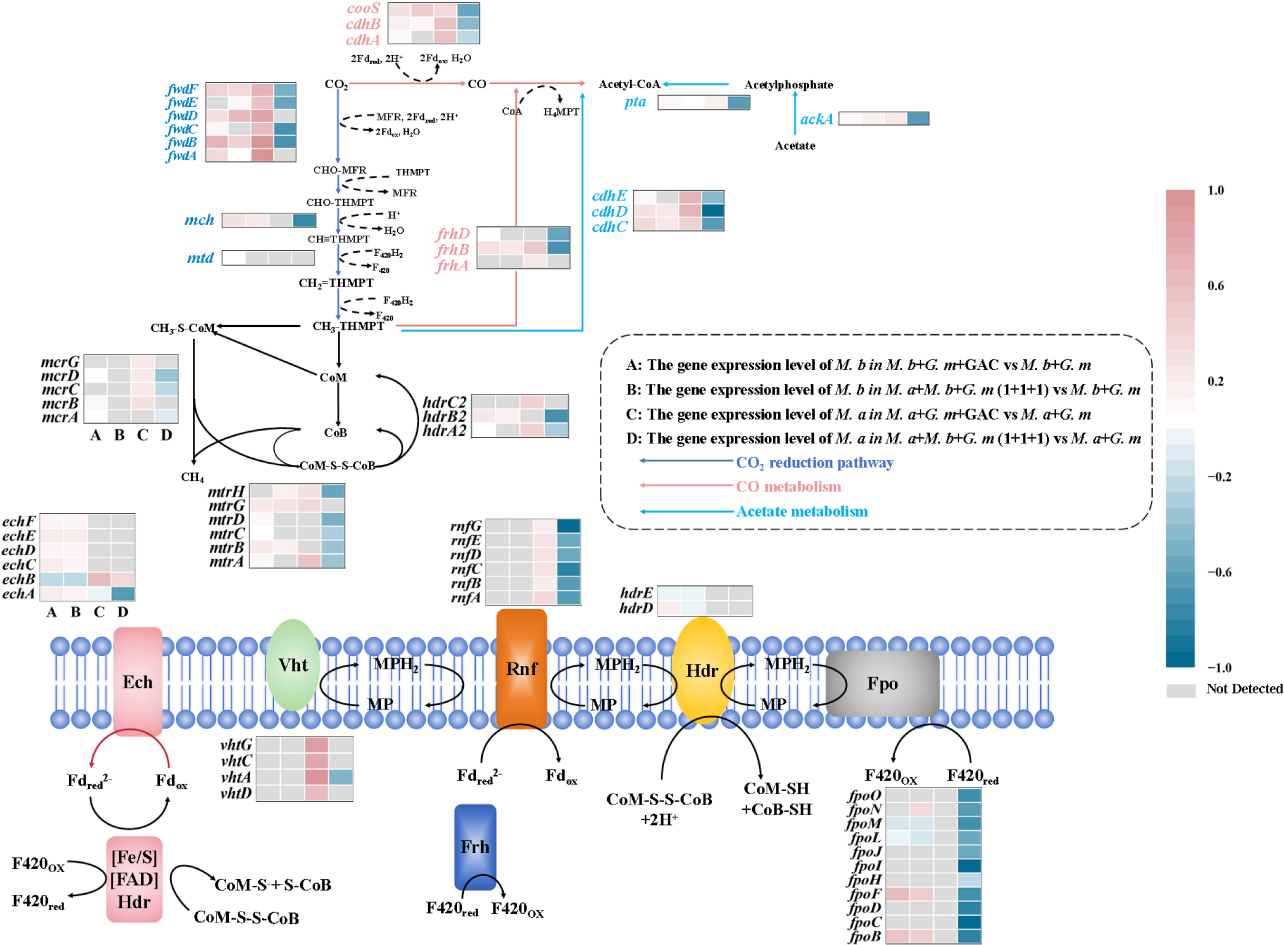
Transcriptomic analysis: Differential expression of carbon metabolism genes. Arrows represent fold up or down regulation of various genes, and only statistically significant values are presented (*p*-values < 0.05). Further details regarding fold differences and *p*-values are available in Supplementary Interactive Plot Data.

After 35 days of cultivation, the same method as that used in the initial inoculation stage was adopted to analyze the relative abundances of the three strains in DM-G systems. The results showed that in the 1:1:1 system, the relative abundances of ASV of *M. acetivorans*, *M. barkeri* and *G. metallireducens* were 0.6%, 39.7% and 59.8%, respectively. In the 1:1:2 system, the relative abundances of *M. acetivorans*, *M. barkeri* and *G. metallireducens* were 0.2%, 35.4% and 64.4%, respectively (Figure 2). Meanwhile, to verify the existence of aggregates, FISH analysis was also conducted on the two DM-G co-culture systems after 35 days of cultivation (Figure 3). *G. metallireducens* cells appeared as yellow rods, while *M. barkeri* cells were green spheres and *M. acetivorans* cells were red spheres. In aggregates, it was difficult to differentiate between individual *Methanosarcina* cells because when the signals overlapped, they turned a blue-violet hue. This made it difficult to use FISH to analyze the relative proportions of *Methanosarcina*. However, it could be concluded that in regions that appeared blue-violet, cells were in close contact with each other (Supplementary Figure 3). These findings indicate that the higher the inoculation amount of *G. metallireducens* in two DM-G co-culture systems.

### 3.3 Gene expression changes induced by conductive material-enhanced SM-G co-culture systems

Transcriptomic comparisons were made between co-cultures with *M. acetivorans* and *G. metallireducens* or *M. barkeri* and *G. metallireducens* in the presence or absence of GAC.

For the GAC-amended *M. barkeri* co-culture, 901 genes were significantly up-regulated (log2FC > 2, *p* < 0.05) and 210 genes were significantly down-regulated (log2FC < −2, *p* < 0.05) (Supplementary Figure 4A). Analysis of the GAC-amended *M. acetivorans* co-culture revealed that 3,483 genes were significantly up-regulated (log2FC > 2, *p* < 0.05) and 123 genes were down-regulated (log2FC < −2, *p* < 0.05) (Supplementary Figure 4B).

Kyoto encyclopedia of genes and genomes (KEGG) metabolic pathway analysis showed that many of the highly expressed genes in *M. barkeri* were related to methane metabolism, redox proteins, cytochrome synthesis and sulfur metabolism. For example, transcripts from *cydB*, which encodes cytochrome bd ubiquinol oxidase subunit II and *cysH*, which encodes a protein involved in sulfur assimilation were 21 and 64-times more highly expressed in GAC-amended cells (Supplementary Figure 5A). Several genes from the CO_2_ reduction pathway were also significantly up-regulated in the GAC-amended co-cultures, including formylmethanofuran dehydrogenase subunits (*fwdABCDF*), methylenetetrahydromethanopterin dehydrogenase (*mtd*) and methenyltetrahydromethanopterin cyclohydrolase (*mch*). During the co-culture process, *M. barkeri* utilizes the produced acetate. In the acetate metabolism pathway, the genes *cdhCDE* (acetyl-CoA decarbonylase/synthase), *pta* (phosphate acetyltransferase), and *ackA* (acetate kinase) were all up-regulated. Additionally, the expression levels of *cdhAB*, the genes encoding carbon monoxide dehydrogenase that catalyze the conversion of carbon dioxide to carbon monoxide, were more than 1.6-fold higher in the co-culture system compared to the system without conductive materials. The up regulation of cytochrome synthesis related genes, and the activation of sulfur metabolism genes may promote the synthesis of coenzyme M (CoM) and coenzyme B (CoB), providing substrates for heterodisulfide reductase (Hdr). At the same time, the coordinated up-regulation of methane metabolism genes (such as *cdhC* and *mtaC*) indicates an improved coupling efficiency between the acetyl CoA pathway and the methyl transfer pathway, further driving the flow of carbon toward methane synthesis. The results of this study are consistent with the previously reported mechanism of granular activated carbon promoting DIET (Dang et al., 2016), that is, conductive materials enhance the efficiency of interspecies electron transfer by constructing electron transfer pathways (Figure 3).

Key methanogenesis-related genes were strongly induced, particularly those involved in the CO_2_ reduction pathway, formylmethanofuran dehydrogenase subunits (*fwdABCDEF*); the general methanogenesis pathway (tetrahydromethanopterin S-methyltransferase, *mtrA*); the CO metabolism related metabolic pathway genes were significantly upregulated, including the genes *cdhAB* (acetyl-CoA decarbonylase/synthase), *cooS* (anaerobic carbon-monoxide dehydrogenase catalytic subunit) and *frhABD* (coenzyme F420 hydrogenase subunit) and the acetate metabolism (acetyl-CoA decarbonylase/synthase *cdhCDE*). Concurrently, genes critical for electron transfer processes exhibited marked activation, including the Rnf complex (*rnfABCDEG*) for DIET and hydrogenases (*frhABD*, *vhtACDG*) for hydrogen cycling. These findings collectively highlight the coordinated metabolic and electron transport adaptations driving enhanced syntrophic activity in GAC-amended systems (Figure 3) These findings suggest that GAC-mediated DIET boosts carbon fixation in *M. acetivorans*. This might be due to better intracellular redox balance and energy conservation, ramping up carbon assimilation for methanogenesis. This aligns with prior studies on conductive material-mediated DIET (Wang et al., 2024). The notable upregulation of redox-related genes implies GAC alters intracellular redox balance, potentially maintaining methane-metabolism enzyme activity, as these enzymes often need a particular redox environment to work well (Supplementary Figure 5B).

This indicates that GAC amendments enhanced methanogenesis via the CO_2_ reduction pathway and increased expression of proteins involved in the maintenance of cellular redox homeostasis. The significant up-regulation of genes related to redox regulation indicates that the intracellular redox balance may have been altered in the presence of GAC.

**FIGURE 4:**
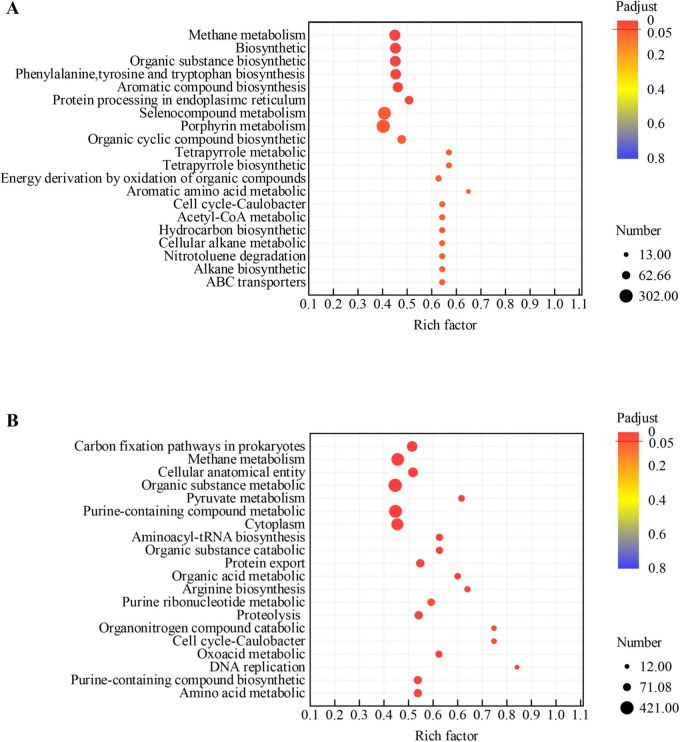
Functional enrichment analysis of DEGs in co-culture systems. Rich factor indicates the ratio of DEGs in a pathway to the total annotated genes in that pathway. Padjust denotes the Benjamini-Hochberg adjusted *p*-value. **(A)** GO enrichment analysis for the DEGs between the *M. barkeri*+*G. metallireducens*+GAC and *M. barkeri*+*G. metallireducens* co-culture systems; **(B)** GO enrichment analysis for the DEGs between the *M. acetivorans*+*G. metallireducens*+GAC and *M. acetivorans*+*G. metallireducens* co-culture systems.

Based on GO enrichment analysis, methane metabolism, biosynthetic, organic substance biosynthetic pathways were enriched in GAC-amended *M. barkeri* DIET co-cultures (Figure 4A). This result indicates that the introduction of GAC had a stimulatory effect on the expression of genes related to carbon metabolism and the biosynthesis of cellular material. Similarly, in the GAC-amended *M. acetivorans* co-culture, many genes coding for proteins involved in carbon fixation via the reductive acetyl-CoA pathway and methane metabolism via the CO_2_ reduction pathway were highly expressed (Figure 4B). Among them, the enrichment of the carbon fixation pathway highlights the enhanced ability of *M. acetivorans* to fix inorganic carbon such as CO_2_. It realizes the conversion of carbon sources into cellular biomass through pathways, providing substrate support for methane metabolism. The enhancement of the methane metabolism pathway directly improves its methane-producing efficiency. These results highlight the regulation of the core pathways of carbon fixation and methane metabolism, directly enhancing the utilization efficiency of carbon sources.

### 3.4 Gene expression changes induced by DM-G co-culture

Transcriptomic comparisons were also made between the dual *Methanosarcina* and *G. metallireducens* co-culture system (*M. acetivorans*+*M. barkeri*+*G. metallireducens*, 1:1:1) and the SM-G control groups. The 1:1:1 ratio was chosen for its optimal methane production (Figure 1A), ecological balance, and robust identification of DIET-associated DEGs, enabling comparative analysis of metabolic complementarity between dual and single *Methanosarcina* systems. A total of 799 genes were up-regulated (log2FC > 2, *p* < 0.05) and 297 genes were down regulated (log2FC < −2, *p* < 0.05) in *M. barkeri* within the DM-G co-culture system (Supplementary Figure 6A).

The most highly expressed genes coded for proteins involved in methane metabolism, pyruvate metabolism, coenzyme A biosynthesis, and carbon fixation. Some of the most highly expressed methane metabolism genes included formylmethanofuran dehydrogenase (*fwdBDF*) and methenyltetrahydromethanopterin cyclohydrolase (*mch*), from the CO_2_ reduction pathway and tetrahydromethanopterin S-methyltransferase (*mtrBGH*) from the general methanogenesis pathway. Additionally, several genes involved in stress response such as hydroxyacylglutathione hydrolase (*gloB*), carbon metabolism such as NADP^+^-dependent malic enzyme (*sfcA*), and amino acid biosynthesis such as acetolactate synthase (*ilvB*) were significantly up regulated. In addition, acetate metabolism genes including *cdhCD* (acetyl-CoA decarbonylase/synthase) and *ackA* (acetate kinase) were 4.6 and 3.5 times more highly expressed in the DM-G system than the SM-G system (Figure 3, Supplementary Figure 7A). These results suggest that the tri-culture system was conducive to the growth of *M. barkeri*.

Contrary to results from analysis of the *M. barkeri* transcriptome, the tri-culture system had an inhibitory effect on expression of many of the genes in *M. acetivorans*. A total of 1,366 genes were significantly down regulated (log2FC < −2, *p* < 0.05), while only 63 genes were up regulated (log2FC > 2, *p* < 0.05) (Supplementary Figure 6B).

Key genes coding for proteins involved in methanogenesis, such as *cdhA*, *frhB*, *fwdB*, *hdrD*, and *mtrA* were all significantly down regulated (Supplementary Figure 7B). *M. acetivorans* also had significantly lower expression of *cdhCDE* (acetyl-CoA decarbonylase/synthase) and *ackA* (acetate kinase) from the acetoclastic pathway in the DM-G system compared to the SM-G system. The *rnfABCDEG* gene cluster, which encodes a sodium-translocating electron transport complex essential for aceticlastic methanogensis (Ferry, 2020) and DIET (Holmes et al., 2021) by *M. acetivorans* was also significantly down-regulated in the DM-G system (Figure 3).

**FIGURE 5:**
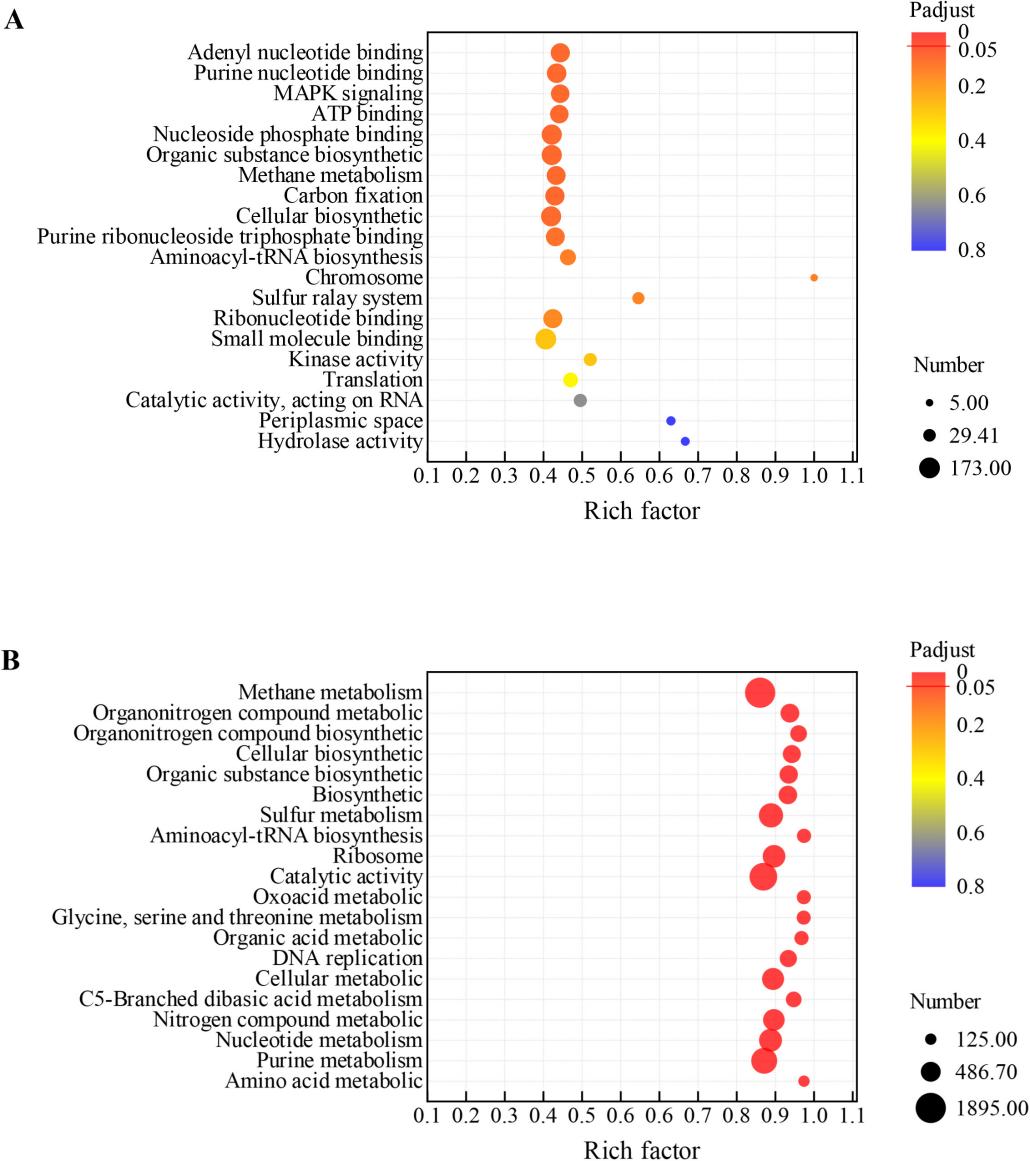
Functional enrichment analysis of DEGs in co-culture systems. Rich factor indicates the ratio of DEGs in a pathway to the total annotated genes in that pathway. Padjust denotes the Benjamini-Hochberg adjusted *p*-value. **(A)** GO enrichment analysis for the DEGs between the *M. acetivorans*+*M. barkeri*+*G. metallireducens* (1+1+1) and *M. barkeri*+ *G. metallireducens* co-culture systems; **(B)** GO enrichment analysis for the DEGs between the *M. acetivorans*+*M. barkeri*+*G. metallireducens* (1+1+1) and *M. acetivorans*+*G. metallireducens* (1+1+1) co-culture systems.

According to GO enrichment analysis, the DEGs of *M. barkeri* are enriched in molecular functions such as Adenyl nucleotide binding, Purine nucleotide binding, and ATP binding, metabolic pathways such as Methane metabolism and Carbon fixation, as well as biological processes such as Cellular biosynthetic process. By strengthening the Methane metabolism pathway, upregulating the expression of key enzyme genes such as methyl coenzyme M reductase, and combining with molecular functions such as ATP binding, it provides support for the energy conversion in the methanogenesis process. At the same time, the Carbon fixation pathway is activated, and inorganic carbon is fixed through pathways such as acetyl coenzyme A, which not only generates cellular biomass but also supplies intermediate substrates for methanogenic metabolism. Moreover, biological processes such as Cellular biosynthetic process are active, ensuring the synthesis of enzyme systems related to methanogenesis and carbon fixation (Figure 5A). In the DM-G systems, the functional enrichment analysis of *M. acetivorans*, shows that core pathways such as methane metabolism and sulfur metabolism are significantly enriched (Figure 5B). However, gene expression confirms that the key genes of these pathways are overall downregulated. Specifically, the efficient capture of electrons by *M. barkeri* creates substrate competition with *M. acetivorans*, directly leading to the suppression of the expression of methane metabolism pathways (such as Methane metabolism and Sulfur metabolism). Essentially, the functional demand of the pathways still exists, but the competition among bacterial populations causes *M. acetivorans* to lose its metabolic dominance. At the same time, the genes of the organic nitrogen compound metabolism pathway are downregulated. This is not an active optimization of carbon-nitrogen synergy, but rather that *M. acetivorans* is forced to reduce its nitrogen metabolic activity when *M. barkeri* competes for nitrogen sources. Moreover, the redox enzyme activity pathway is enriched, which only corresponds to *M. acetivorans* up-regulating a small number of emergency genes to maintain the basic functions of the electron transport chain (such as the Rnf complex and iron-sulfur proteins), but the core energy conversion efficiency (such as ATP production and methane yield) has not been improved.

### 3.5 Comparison of transcriptomes between DM-G co-culture and conductive material-enhanced SM-G co-culture systems

To determine whether similar pathways were induced in the DM-G system and the GAC-amended SM-G co-culture systems, further transcriptomic comparisons were made. The *M. barkeri*+*G. metallireducens*+GAC co-culture system had 467 unique DEGs, while the *M. acetivorans*+*M. barkeri*+*G. metallireducens* (1+1+1) system had 452 unique DEGs, with 644 shared DEGs between them (Supplementary Figure 8A). For the *M. acetivorans*+*G. metallireducens*+GAC and *M. acetivorans*+*M. barkeri*+ *G. metallireducens* (1+1+1) systems, the number of unique DEGs in *M. acetivorans*+*G. metallireducens*+GAC was 262, and in *M. acetivorans*+*M. barkeri*+ *G. metallireducens* (1+1+1) was 2439, with 1167 shared DEGs (Supplementary Figure 8B). These differences in DEGs indicate that there were differences in metabolism between the tri-culture and GAC-amended systems.

**FIGURE 6:**
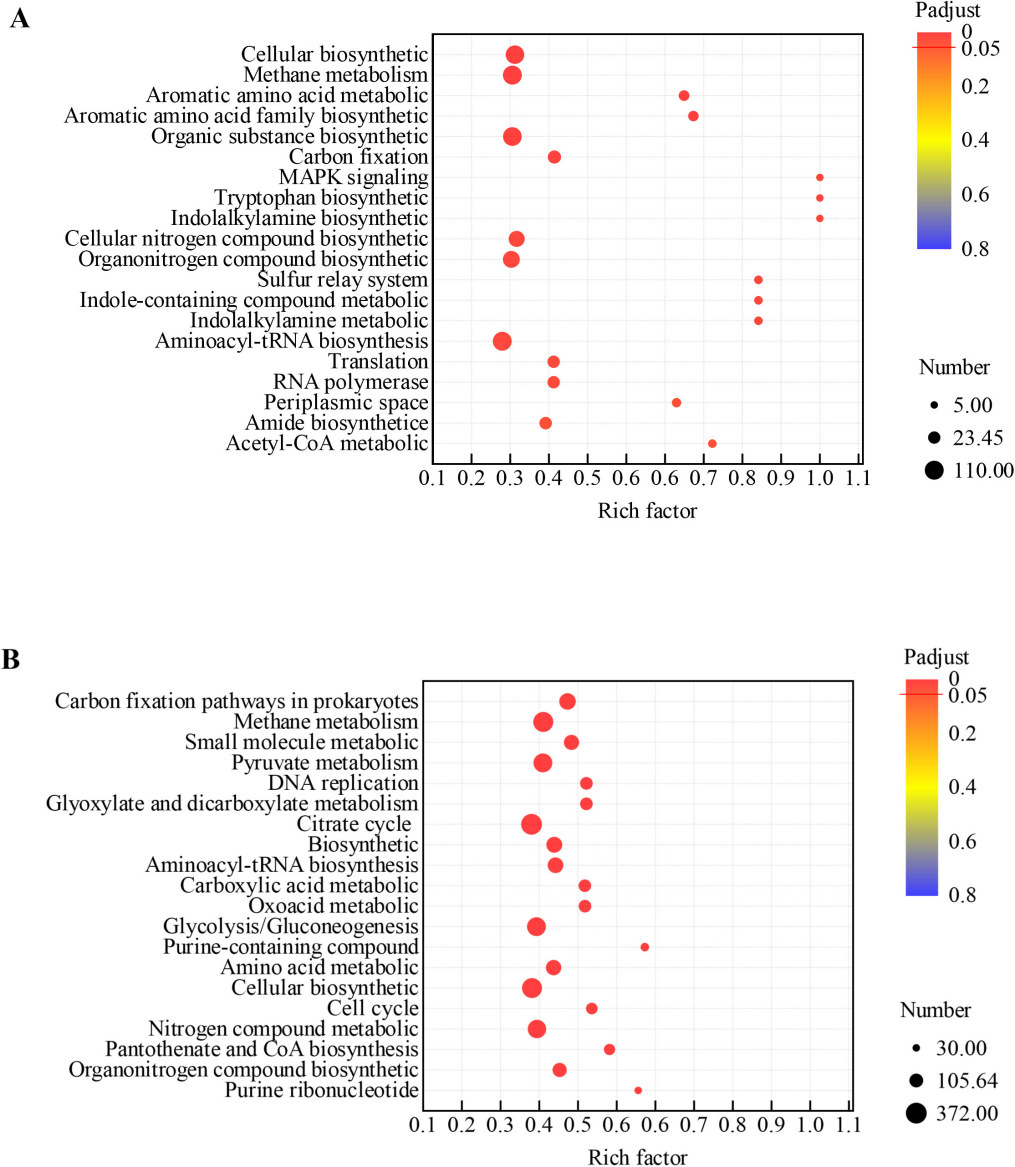
Functional enrichment analysis of DEGs in co-culture systems. Rich factor indicates the ratio of DEGs in a pathway to the total annotated genes in that pathway. Padjust denotes the Benjamini-Hochberg adjusted *p*-value. **(A)** GO enrichment analysis for the DEGs between the *M. barkeri*+*G. metallireducens*+GAC and *M. acetivorans*+*M. barkeri*+*G. metallireducens* (1+1+1) co-culture systems; **(B)** GO enrichment analysis for the DEGs between the *M. acetivorans*+*G. metallireducens*+GAC and *M. acetivorans*+*M. barkeri*+*G. metallireducens* (1+1+1) co-culture systems.

Gene enrichment analysis revealed that genes involved in cellular biosynthesis, aromatic amino acid biosynthesis, and methane metabolism were highly expressed by *Methanosarcina* in both enhanced systems (Figure 6A). The up-regulation of these biosysnthesis genes supports the finding that cells were growing better in the enhanced systems. Methane metabolism was the most prominently enriched pathway, but carbon fixation pathways were also significantly enriched. The concurrent enrichment of small molecule metabolism, pyruvate metabolism, and glycolysis/gluconeogenesis pathways suggests that cells were growing and generating biomass (Figure 6B). Furthermore, carbon fixation pathways were significantly enriched in both *M. barkeri*+*G. metallireducens*+GAC and *M. acetivorans*+*M. barkeri*+*G. metallireducens* (1:1:1), indicative of enhanced carbon assimilation and its integration into biomass synthesis, potentially accelerating substrate turnover (Figure 6A).

The enrichment analysis of DEGs in the *M. acetivorans*+*G. metallireducens*+GAC and *M. acetivorans*+*M. barkeri*+*G. metallireducens* (1:1:1) identified methane metabolism as the most significantly enriched pathway. This result highlights the conserved role of methanogenesis-related genes across different microbial consortia. The concurrent enrichment of small molecule metabolism, pyruvate metabolism, and glycolysis/gluconeogenesis pathways suggests a metabolic shift toward core energy conservation and biosynthetic processes, which are essential for microbial growth and proliferation. These enriched pathways collectively enhance ATP production and the redistribution of carbon flux, thereby sustaining cellular growth and facilitating methanogenic activity (Figure 6B).

## 4 Discussion

This study systematically investigated the synergy within DM-G co-culture systems for enhancing DIET and methane production. The results provide novel insights into microbial competition dynamics, metabolic pathway regulation, and the functional implications of DIET-driven syntrophy.

The DM-G co-culture systems demonstrated marked improvements in DIET efficiency and methane yield compared to SM-G systems. Mechanistically, *M. acetivorans* exhibited slower acetate oxidation due to its lack of hydrogenase activity, leading to elevated acetate accumulation. In contrast, *M. barkeri* efficiently utilized electrons from *G. metallireducens* via hydrogenase-mediated pathways, accelerating acetate conversion to methane through hydrogen cycling (Zhou et al., 2021). Two *Methanosarcina* species may achieve efficient electron transfer and utilization through distinct pathways or mechanisms, compensating for the limitations of a single *Methanosarcina* and enhancing DIET efficiency. In terms of metabolic pathways, the synergistic effect of *M. barkeri* and *M. acetivorans* may have created new metabolic pathways or optimized existing ones, allowing ethanol to be converted into methane more quickly and thoroughly, thereby improving substrate utilization efficiency. This divergence in metabolic strategies highlights the complementary roles of the two *Methanosarcina* in optimizing substrate utilization and electron flux regulation during syntrophic ethanol oxidation (Schöne et al., 2023).

Microbial community analysis revealed that in the DM-G co-culture system, as the cultivation time extended, *M. barkeri* gradually became dominant, while the relative abundance of *M. acetivorans* gradually decreased. This change in the community structure indicates that under high electron flux conditions, *Methanosarcina* with hydrogenase may have a greater competitive advantage. Such ecological succession may redirect the metabolic flux toward hydrogenotrophic methanogenesis, thereby altering the stability of the system and the substrate utilization patterns (Zhou et al., 2021). Additionally, the persistent presence of *G. metallireducens* throughout the cultivation process demonstrates its crucial role in maintaining the function of DIET despite changes in the community structure (Thauer et al., 2008). These findings highlight the necessity of evaluating the long-term stability of syntrophic relationships in engineered systems.

Metabolic pathway analysis revealed the metabolic interaction mechanisms in the co-culture system of dual *Methanosarcina* and *G. metallireducens*. In the DM-G system, the gene expression of *M. barkeri* showed a significant up-regulation trend, especially the high expression of genes related to acetate metabolism and methane production (such as *cdhCD* and *ackA*), indicating that it had a functional advantage in acetate utilization and could efficiently convert acetate into methane. This phenomenon might be attributed to the introduction of *M. acetivorans*, which intensified the electron competition and promoted niche differentiation, prompting *M. barkeri* to strengthen the acetate metabolic pathway. In contrast, the gene expression of *M. acetivorans* was generally inhibited. The core genes of its methane metabolism (*cdhA*, *frhB*) and genes related to carbon fixation (*mrtF*) were significantly downregulated, but the methane production pathway dependent on electron transfer remained active (Figure 3). Specifically, the expression pattern of *rnfABCDE* cluster encoding the Rnf electron transfer complex in the co-culture system showed that the electron transfer process was regulated by the microbial interaction environment, suggesting that it obtained exogenous electrons through interaction with *G. metallireducens* and shifted to the synthesis of methane via the carbon dioxide reduction pathway. The above results are consistent with the previous research conclusions, *M. acetivorans* is more efficient in methane production dependent on DIET, while *M. barkeri* exhibits stronger aceticlastic methanogenic activity (Zhou et al., 2021). Through metabolic pathway differentiation (acetate decomposition and electron-dependent CO_2_ reduction) and niche complementarity, the two bacteria form a synergistic metabolic network in the conductive material-mediated co-culture system, ultimately improving the methanogenic efficiency.

From an application perspective, DM-G co-culture systems offer significant advantages over conductive material-based approaches. By eliminating exogenous material requirements, these systems avoid associated economic costs and environmental risks (de Bok et al., 2004; Wu B. et al., 2020). The natural metabolic coupling between *Methanosarcina* species and *G. metallireducens* provides a cost-effective and sustainable alternative that maintains process stability while reducing reliance on external inputs (McInerney et al., 2008). DM-G co-culture systems demonstrate scalability potential for biogas production, combining operational simplicity with environmental compatibility–key factors for industrial adoption (Bochuan et al., 2014; Thakur et al., 2022).

## 5 Conclusion

This study explored the enhancement of methane production through co-culturing two *Methanosarcina* species (*M. barkeri* and *M. acetivorans*) and *G. metallireducens* during ethanol consumption. The DM-G co-culture systems demonstrated efficient methane production without the need for exogenous conductive materials. Transcriptomic analysis revealed that the synergistic interaction between the two *Methanosarcina* species optimized electron transfer and metabolic pathways, leading to enhanced methane production. *M. barkeri* exhibited a competitive advantage, with upregulated genes related to methane metabolism and electron transfer, while *M. acetivorans* displayed downregulation of methanogenic pathway genes, indicating metabolic complementarity. The DM-G co-culture system offers a sustainable and cost-effective alternative for bioenergy production, with potential applications in organic waste treatment and renewable energy generation.

## Data Availability

The raw data supporting the conclusions of this article will be made available by the authors, without undue reservation.
