# Supplementary material for: Enhancement of direct interspecies electron transfer and methane production by co-culture of dual Methanosarcina species and Geobacter metallireducens
Source: Front Microbiol. 2025 Aug 5;16:1604265. doi: 10.3389/fmicb.2025.1604265 (PMC12392111; doi:10.3389/fmicb.2025.1604265)
Supplement: Supplementary file 1 [file Data_Sheet_1.docx]

Supplementary Material

# Supplementary Figures
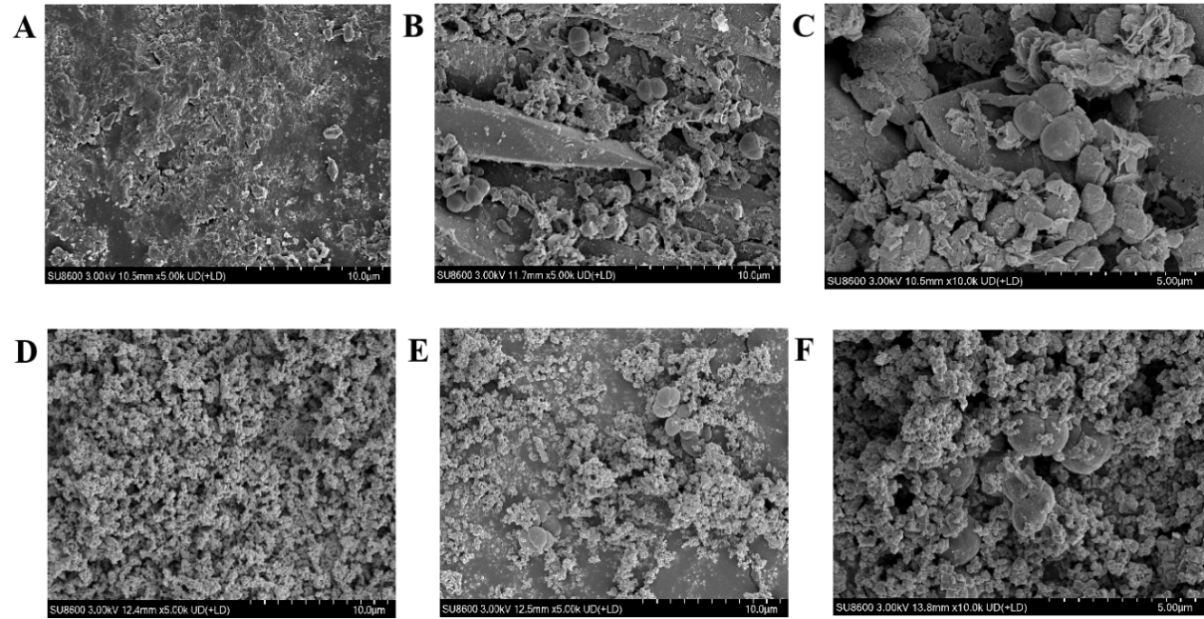


**Supplementary Figure 1.** Scanning Electron Microscope Images: **(A)** 5000×GAC in the blank group; **(B)** 5000×coculture enhanced by GAC; **(C)** 10000×coculture enhanced by GAC; **(D)** Nano-magnetite in the blank group at 5000×magnification; **(E)** Coculture enhanced by nano-magnetite at 5000×magnification; **(F)** Coculture enhanced by nano-magnetite at 10000×magnification.


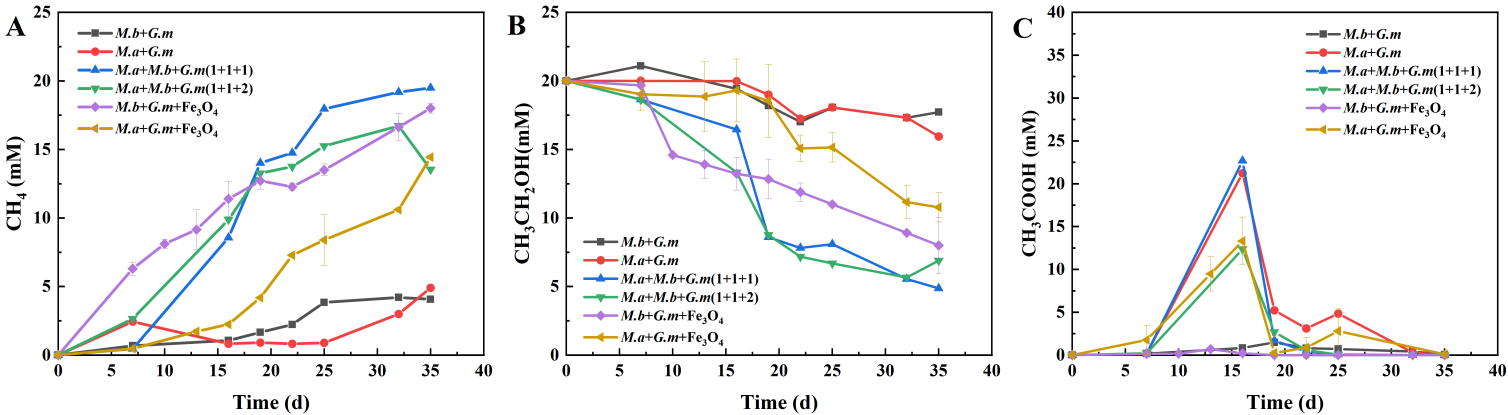


**Supplementary Figure 2.** Methane production, ethanol metabolism, and acetic acid metabolism in SM-G and DM-G co-culture systems with or without nano-magnetite. **(A)** Methane production. **(B)** Ethanol metabolism. **(C)** Acetic acid metabolism. Data are presented as mean values with standard errors from three replicate cultures. Error bars represent the range of standard errors.


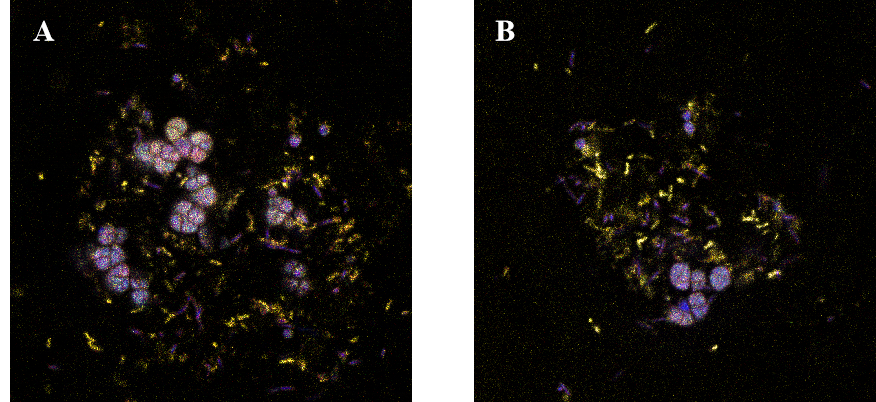


**Supplementary Figure 3.** Spatial distribution characteristics of the microbial community in the DM-G co-culture system based on FISH. **(A)** Distribution in the 1:1:1 system (Day 35); **(B)** Distribution in the 1:1:2 system (Day 35). *G. metallireducens* (Cy5-labeled, far-yellow fluorescence) is shown as rod-shaped cells; *M. barkeri* (FAM-labeled, green fluorescence) and *M. acetivorans* (Cy3-labeled, red fluorescence) are shown as spherical cells. Due to spatial overlap, the merged fluorescence of the two methanogens appears blue-violet. Scale bars=1 μm.


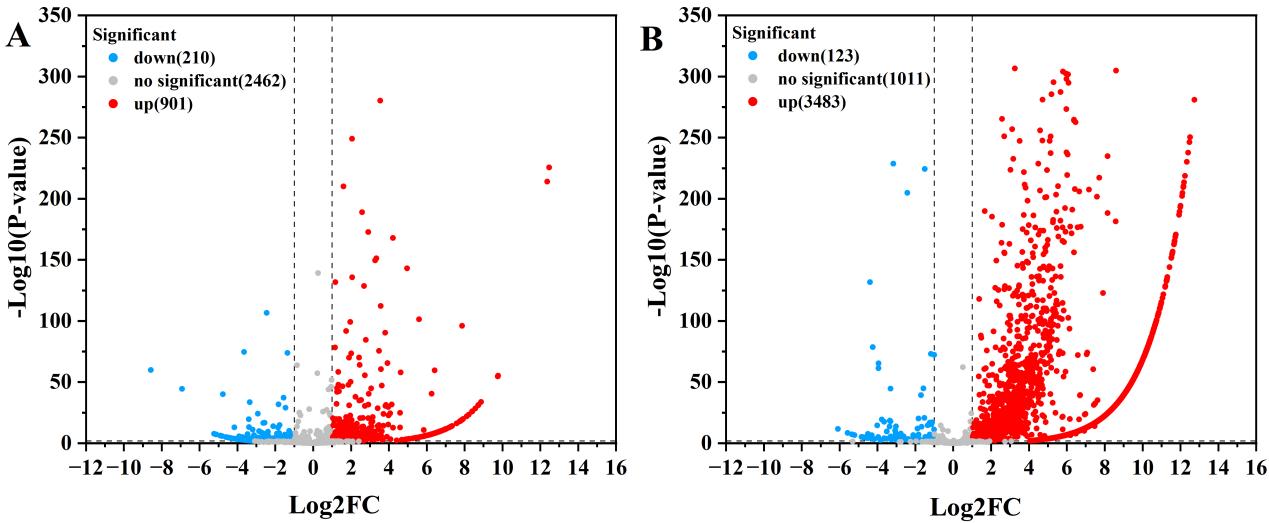


**Supplementary Figure 4.** Volcano plots showing the DEGs of *Methanosarcina* induced by the addition of GAC. (A) DEGs of *M. barkeri* in the *M. barkeri*+*G. metallireducens*+GAC group (*M. barkeri*+*G. metallireducens* was used as a control); (B) DEGs of *M. acetivorans* in the *M. acetivorans*+*G. metallireducens*+GAC group (*M. acetivorans* +*G. metallireducens* was used as a control).


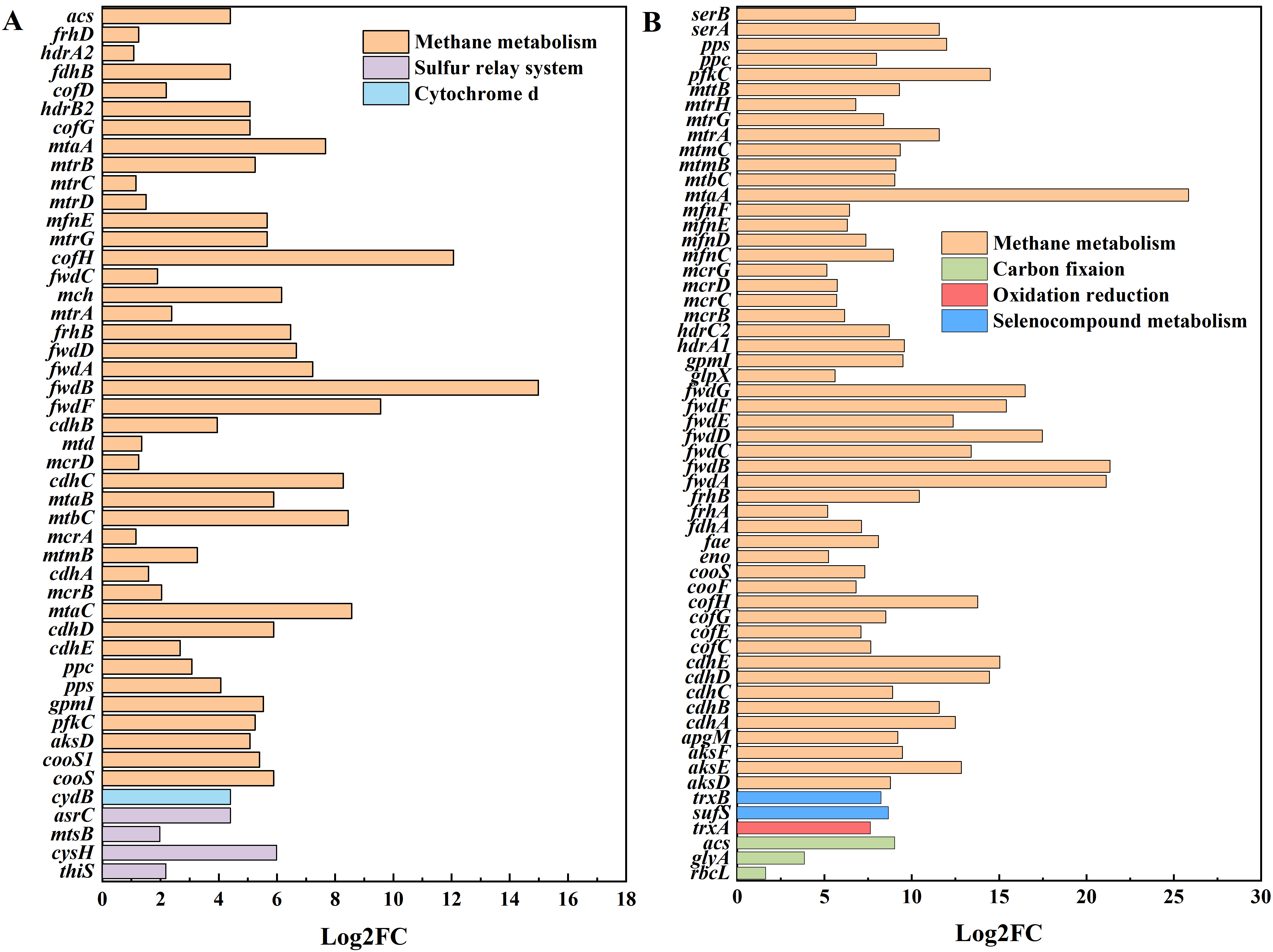


**Supplementary Figure 5.** Gene expression changes induced by the addition of conductive materials (GAC) in *Methanosarcina* and *Geobacter* co-culture systems. **(A)** Log2FC values of representative genes in *M. barkeri* + *G. metallireducens* + GAC compared to *M. barkeri* + *G. metallireducens* (control); **(B)** Log2FC values of representative genes in *M. acetivorans* + *G. metallireducens* + GAC compared to *M. acetivorans* + *G. metallireducens* (control).


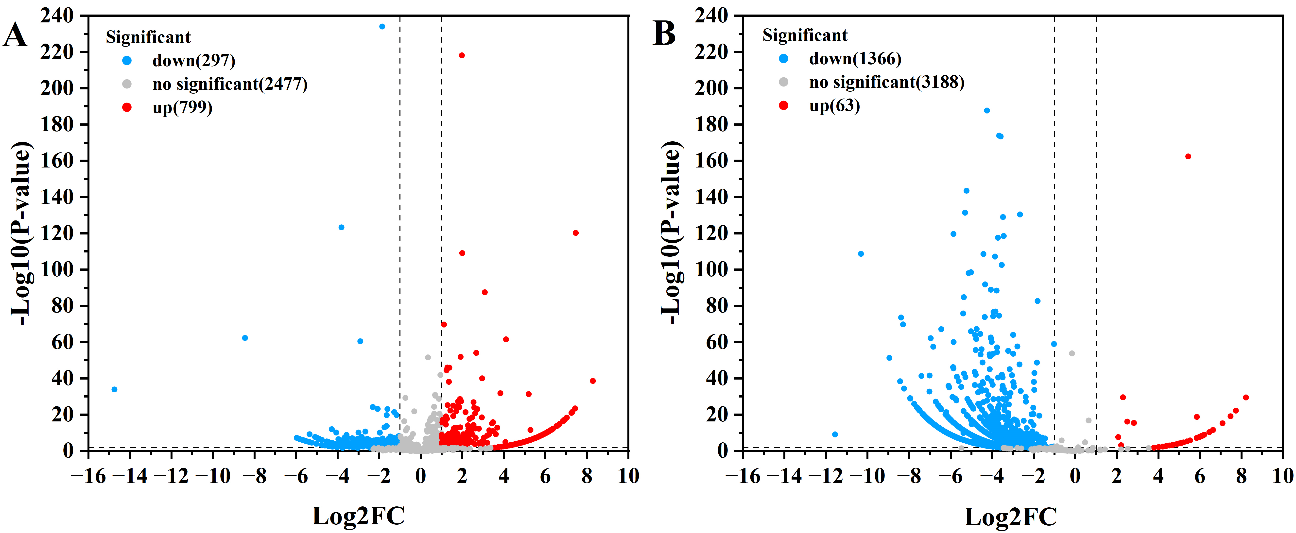


**Supplementary Figure 6.** Volcano plots showing the DEGs of *Methanosarcina* in the DM-G system. (A) Transcription of all genes in the DM-G system (*M. barkeri*+*G. metallireducens* was used as a control); (B) Transcription of all genes in the DM-G system(*M. acetivorans* +*G. metallireducens* was used as a control).


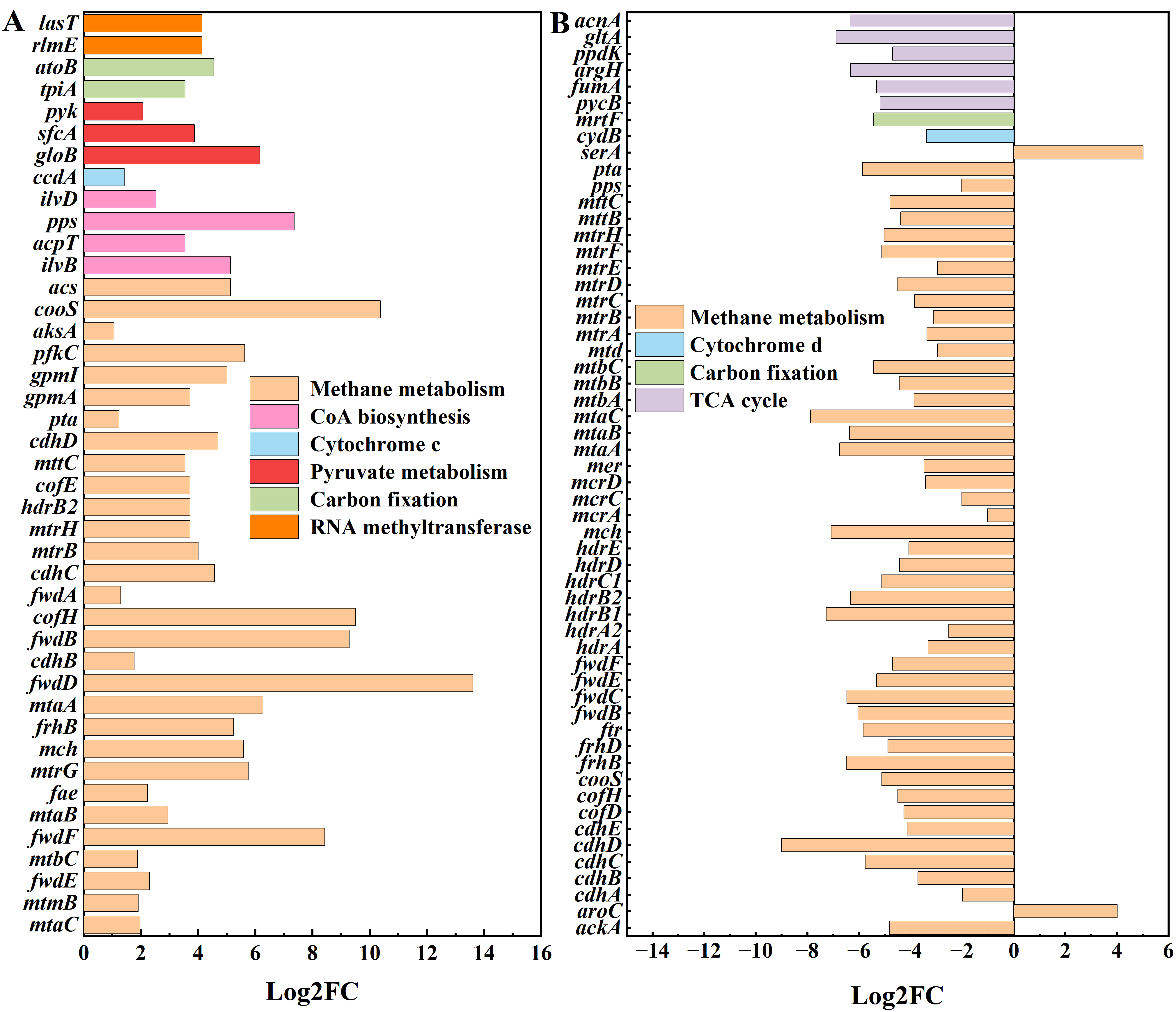


**Supplementary Figure 7.** Gene expression changes induced by dual *Methanosarcina* co-culture with *G. metallireducens*. **(A)** Log2FC values of representative genes in *M. acetivorans* + *M. barkeri* + *G. metallireducens* (1+1+1) compared to *M. barkeri* + *G. metallireducens* (control); **(B)** Log2FC values of representative genes in *M. acetivorans* + *M. barkeri* + *G. metallireducens* (1+1+1) compared to *M. acetivorans* + *G. metallireducens* (control).


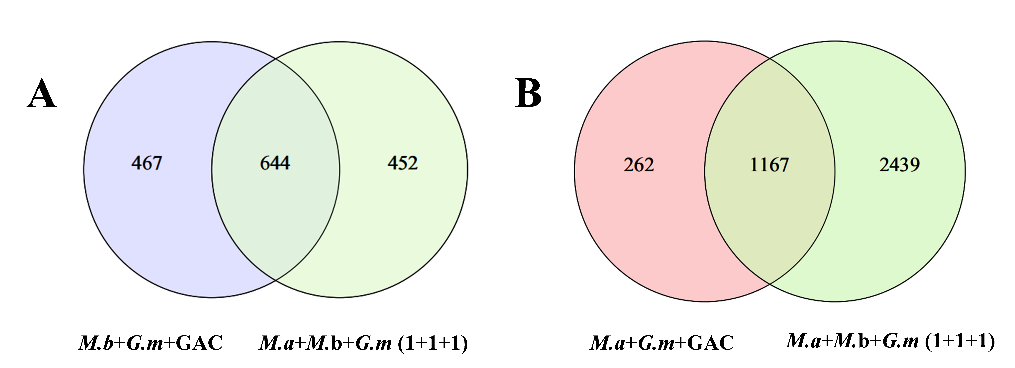


**Supplementary Figure 8.** Venn diagram showing the overlaps of DEGs between different comparison groups. (A) The distribution of DEGs between the co-culture systems of *M. barkeri*+*G. metallireducens*+GAC and *M. acetivorans*+*M. barkeri*+*G. metallireducens* (1+1+1). (B) The distribution of DEGs between the co-culture systems of *M. acetivorans*+*G. metallireducens*+GAC and *M. acetivorans*+*M. barkeri*+*G. metallireducens* (1+1+1).
